# Microglia contact induces synapse formation in developing somatosensory cortex

**DOI:** 10.1038/ncomms12540

**Published:** 2016-08-25

**Authors:** Akiko Miyamoto, Hiroaki Wake, Ayako Wendy Ishikawa, Kei Eto, Keisuke Shibata, Hideji Murakoshi, Schuichi Koizumi, Andrew J. Moorhouse, Yumiko Yoshimura, Junichi Nabekura

**Affiliations:** 1 Division of Homeostatic Development, National Institute for Physiological Sciences, Okazaki 444-8585, Japan; 2 Core Research for Evolutional Science and Technology, Japan Agency for Medical Research and Development, Tokyo 100-0004, Japan; 3 Department of Physiological Sciences, The Graduate School for Advanced Study, Hayama 240-0193, Japan; 4 Precursory Research for Embryonic Science and Technology, Japan Science and Technology Agency, Saitama 102-0076, Japan; 5 Division of Visual Information Processing, National Institute for Physiological Sciences, Okazaki 444-8585, Japan; 6 Department of Pharmacology, Graduated School of Medical and Engineering, Yamanashi University, Chuo 409-3898, Japan; 7 Section of Multiphoton Neuroimaging, National Institute for Physiological Sciences, Okazaki 444-8585, Japan; 8 School of Medical Sciences, The University of New South Wales, Sydney, New South Wales 2052, Australia

## Abstract

Microglia are the immune cells of the central nervous system that play important roles in brain pathologies. Microglia also help shape neuronal circuits during development, via phagocytosing weak synapses and regulating neurogenesis. Using *in vivo* multiphoton imaging of layer 2/3 pyramidal neurons in the developing somatosensory cortex, we demonstrate here that microglial contact with dendrites directly induces filopodia formation. This filopodia formation occurs only around postnatal day 8–10, a period of intense synaptogenesis and when microglia have an activated phenotype. Filopodia formation is preceded by contact-induced Ca^2+^ transients and actin accumulation. Inhibition of microglia by genetic ablation decreases subsequent spine density, functional excitatory synapses and reduces the relative connectivity from layer 4 neurons. Our data provide the direct demonstration of microglial-induced spine formation and provide further insights into immune system regulation of neuronal circuit development, with potential implications for developmental disorders of immune and brain dysfunction.

## Abstract (web summary)

Microglia contribute to shaping neural circuits in the developing brain. Here, the authors show that microglial contact with pyramidal neuron dendrites induces synapse formation in the developing somatosensory cortex, and ablation of microglia reduces synaptic connections from L4 to L2/3 neurons.

## Graphical abstract

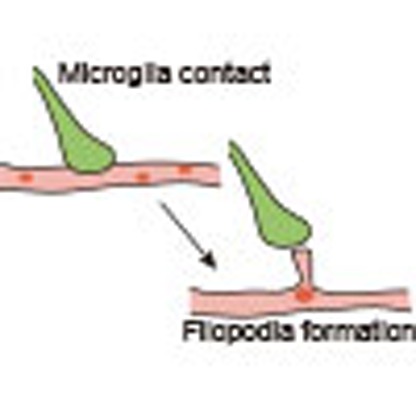

Increasing evidences point to the important influence immune status and immune molecules have on brain development and function^1^. Maternal infection is a considerable risk factor for the neurodevelopmental disorders such as autism and schizophrenia^2^. Microglia are important components of such immune–brain interactions and defects in microglial genes or signalling molecules are associated with disorders of neuronal circuits and brain behaviour^1^,^3^,^4^,^5^. As the immune cells of the central nervous system, microglia respond to various brain pathologies, adopt an activated phenotype and secrete a range of cytokines and neuro-trophic factors to modify disease progression^6^,^7^,^8^,^9^. However, microglia also play important roles for normal brain physiology, both in development and in the mature nervous system^10^. In the developing brain, they actively participate in sculpting neuronal circuits^11^. This includes promoting neuronal apoptosis^12^, regulating neurogenesis^13^,^14^,^15^ and eliminating less active synapse structures identified for phagocytosis via expression of traditional immune complement molecules^10^,^16^,^17^,^18^,^19^,^20^. More recent studies have suggested microglia have bidirectional effects on synapses and neuronal circuits by promoting synapse formation^21^. Parkhurst *et al*.^22^ demonstrated that selective ablation of microglia reduced spine formation as observed by time-lapse imaging *in vivo*, both during later development and in response to different learning tasks. Selective depletion of brain-derived neurotrophic factor (BDNF) from microglia could largely replicate this observation. A role for microglia in enhancing spine density has also been suggested from *in vitro* studies. Addition of microglial cells to hippocampal neuronal cultures could increase the number of dendritic spines via release of interleukin-10 (IL-10)^23^, whereas the effect of estradiol to promote increased spine density in cultured pre-optic neurons depends on the presence and function of microglia^24^. Release of cytokines such as IL-10 by microglia is typically associated with an activated microglia phenotype, such as occurs when microglia begin to populate the cortex during early postnatal development. However, (1) how microglia may contribute to functional synapse formation; (2) whether this is a direct result of microglia–neuron interactions; and (3) the functional significance of putative microglia-induced spine formation in development is all unclear. To address these questions, we undertook in this study *in vivo* two-photon time-lapse imaging of neurons and microglia in the developing somatosensory cortex. We observed that microglia directly contacted dendrites and initiated filopodia formation. This effect of microglia was restricted to the period of intense spinogenesis that occurs in the first two postnatal weeks, a time when microglia have an activated morphological phenotype. We propose that filopodia formation by microglia promotes the maturation of specific neuronal circuit connections through the formation of functional mature synapses.

## Results

### Microglia promote filopodia formation during development

**Figure 1:**
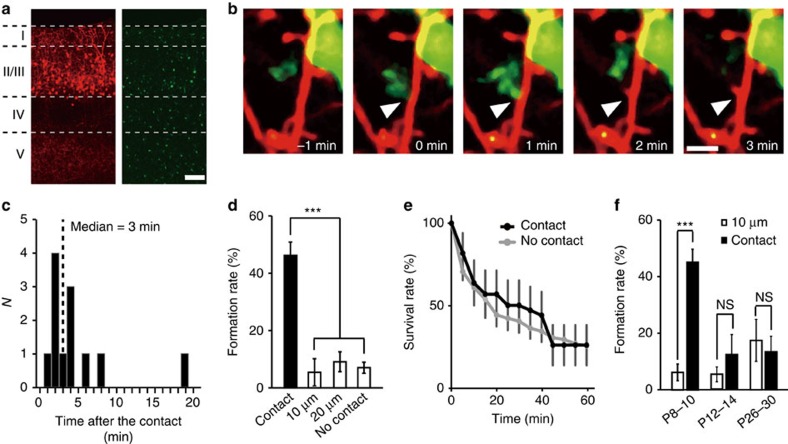
Microglia initiate filopodia formation in developing somatosensory cortex. (**a**) Fluorescent image of P10 somatosensory cortex showing the distribution of L2/3 neurons (tdTomato, red) and microglia (EGFP, green), Scale bar, 50 μm. (**b**) Time-lapse *in vivo* images showing dendritic filopodia formation following microglia contact (white arrow indicates contact point, red represents dendrites, green represents microglia). Scale bar, 2 μm. (**c**) Histogram showing the distribution of latencies of filopodia formation following microglia contact (*n*=12 filopodia from 5 animals, bin size=1 min). (**d**) Filopodia formation rate at microglia contact points (contact) and at dendritic regions 10 and 20 μm adjacent to the contact point. ‘No contact' represents the entire dendrite, excluding the contact point (one-way ANOVA, *post-hoc* Bonfferoni test, error bars are mean ±s.e.m.; exact *P*-value is *P*=0.0000002; *n*=8 animals). (**e**) Survival rate of filopodia formed following microglia contact (black line) or formed without prior microglia contact (grey line) (two-way ANOVA, *post-hoc* Bonfferoni test, error bars are mean ±s.e.; exact *P*-value is *P*=0.11; *n*=25 dendrites from 9 animals). (**f**) Comparison of filopodia formation rates in dendrites at microglia contact points (contact) and at dendritic locations 10 μm adjacent to the contact points (10 μm) in three different age groups. Microglial contact-induced filopodia decreases over postnatal development (paired *t*-test, error bars are mean ±s.e.m.; exact *P*-values are *P*=0.0003 (P8–P10), *P*=0.37 (P12–P14), *P*=0.41 (P26–P30); P8–P10: *n*=8 animals, P12–P14: *n*=7 animals, P26–P30: *n*=6 animals).

To visualize microglia–dendrite interactions, we performed *in utero* electroporation of embryonic day (E)14–E15 Iba1-EGFP mice with red fluorescent protein (RFP) (tdTomato). This enabled us to successfully image layer (L) 2/3 pyramidal neurons (tdTomato, red) alongside microglia (enhanced green fluorescent protein (EGFP), green; Fig. 1a). Subsequent time-lapse imaging in postnatal day (P) 8–P10 pups detected microglia–neuron contacts, as we and others had observed in more mature cortex^19^,^20^. However, in the developing somatosensory cortex, microglia–dendrite contacts were frequently followed by the formation of new dendritic filopodia. Typically, filopodia formation occurred within 10 min following microglia contact, with a median latency of 3 min (Fig. 1b,c and Supplementary Movie 1). Filopodia also developed from dendrites without apparent microglial contacts, but the incidence of new filopodia formation in a dendrite was significantly greater at the microglia contact points (45±4.5%), as compared with non-contact regions at dendritic locations 10 μm (6.3±2.9%) and 20 μm (8.9±3.4%) adjacent to the contact point, and as compared with formation rates in the total dendrite, excluding the contact zone (6.9±1.9%; Fig. 1d; one-way analysis of variance (ANOVA), *post-hoc* Bonfferoni test, error bars are mean ±s.e.m.; exact *P*-value is *P*=0.0000002, exact F-value is F(3, 77)=20.2; *n*=8 animals). The typical duration of microglia–dendrite contacts was about 9 min and this contact duration was the same whether contact was followed by filopodia formation (duration of contact 9.0±1.6 min) or was not followed by filopodia formation (9.0±1.4 min; Supplementary Fig. 1, unpaired *t*-test, exact *P*-value is *P*=0.995, exact *t*-value is *t*(50)=0.007, *n*=26 animals). Newly formed filopodia are highly labile^25^ and we observed ∼75% of filopodia disappearing within 60 min, for both filopodia initiated by microglia contact or for those arising independent of contact (Fig. 1e; two-way ANOVA, *post-hoc* Bonfferoni test, exact *P*-value is *P*=0.11, exact F-value is F(3, 77)=20.2; *n*=25 dendrites from 9 animals). Microglia contact-induced filopodia was only observed at P8–P10, when microglia have an activated phenotype and when the total number of spines in L2/3 somatosensory cortex is rapidly increasing^26^. At P12–P14 and P26–P30, microglia still contacted dendrites with a similar frequency, but the rate of subsequent filopodia formation (12.2±7.1 and 13.5±5.5%, respectively) was not significantly different from that at adjacent non-contacted points (Fig. 1f; paired *t*-test, exact *P*-values are *P*=0.0003 (P8–P10), *P*=0.37 (P12–P14) and *P*=0.41 (P26–P30); exact *t*-values are *t*(7)=6.61 (P8–P10), *t*(12)=0.92 (P12–P14) and *t*(10)=0.85 (P26–P30); P8–P10: see Fig. 1d; P12–P14: *n*=7 animals; P26–P30: *n*=6 animals).

### Signalling events underlying microglial-induced filopodia

**Figure 2:**
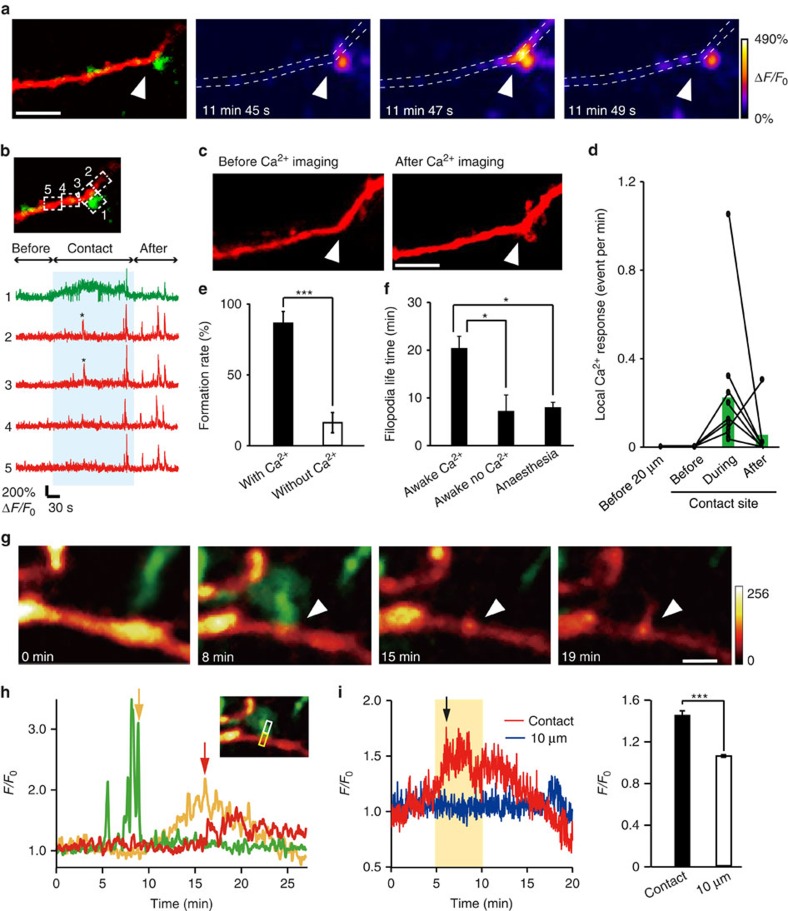
Local dendritic Ca^2+^ elevation and actin accumulation may mediate microglia-induced filopodia formation. (**a**) Ca^2+^ imaging in awake mouse *in vivo*. Left panel shows a dendrite (red) with a microglia (green) contact point indicated by the white arrow head. Subsequent panels show Ca^2+^ fluorescence at different times during the same microglia–dendrite contact. The white dotted line indicates the dendrite outline. (**b**) Upper left panel shows the same dendrite (red) and microglia (green) contact as in **a** and indicates the ROIs used to quantify the spatial and temporal aspects of Ca^2+^ elevation shown in the corresponding five traces in the right panel. The blue shading indicates the duration of the microglia–dendrite contact, which induced brief Ca^2+^ transients (indicated by asterisk) in ROIs 2 and 3, and a smaller sustained elevation in the microglial process (ROI 1). (**c**) *Z*-stacked images of the same dendrite before the Ca^2+^ imaging and transients were observed and following Ca^2+^ imaging. Arrowheads indicate microglia contacted point. Scale bar, 5 μm. (**d**) Local dendritic Ca^2+^ responses were exclusively associated with microglial contact. At dendritic contact sites, Ca^2+^ transients were absent before the contact, increased in frequency during the contact and typically decreased back towards zero after the contact. At dendritic locations 20 μm adjacent to the contact points, no localized Ca^2+^ responses were observed throughout the same imaging periods (20 μm *n*=3, before contact *n*=4, during contact *n*=11, after contact *n*=7 dendrites in 8 mice). (**e**) Averaged filopodia formation rates in dendrites in which microglial contact was associated with a Ca^2+^ transient compared with those dendrites in which there was no Ca^2+^ response-associated microglia contact (unpaired *t*-test, error bars are mean ±s.e.m., exact *P*-value is *P*=0.00006; with Ca^2+^: *n*=7 animals, without Ca^2+^: *n*=16 animals). (**f**) Lifetime of filopodia formed after microglial contact to dendrites in which a Ca^2+^ response was observed and in those in which microglial contact was not associated with a dendritic Ca^2+^ transient. These experiments were conducted in awake mice and are compared with lifetimes of filopodia arising after microglial contact in dendrites of anaesthetized mice (one-way ANOVA, *post-hoc* Bonfferoni test, error bars are mean ±s.e.m., exact *P*-values are *P*=0.002 (awake Ca^2+^, awake no Ca^2+^), *P*=0.0003 (awake Ca^2+^, anaesthesia), *P*=1 (awake no Ca^2+^, anaesthesia); awake Ca^2+^: *n*=7, awake no Ca^2+^: *n*=5, anaesthesia: *n*=16 dendrites). (**g**) Time-lapse *in vitro* imaging of actin accumulation after microglia contact with a dendrite in a cortical slice culture transfected with pCMV-lifeact-mCherry. Arrowhead indicates microglia contact point. mCherry colour intensity scale shown in arbitrary units; green, microglia. Scale bar, 2 μm. (**h**) Quantification of the time course of fluorescence changes for the images illustrated in G (microglia, green; lifeact-mCherry, yellow; filopodia, red). The inset shows the ROI used to quantify fluorescence (white box for microglia process and dendritic filopodia, yellow box for actin accumulation). Yellow arrow indicates end of the microglia contact. Red arrow indicates start of filopodia formation. (**i**) Left: representative time course of normalized lifeact-mCherry fluorescent intensity changes within the dendrite at a microglia contact site (red) and at an adjacent dendritic site 10 μm from the contact point (blue). Black arrow indicates onset of filopodia formation. Right: averaged fluorescent intensity values from 5 to 10 min after microglia contact at contact sites was significantly increased compared with that at adjacent sites 10 μm from the microglia contact points during the same time period (right) (paired *t*-test, error bars are mean ±s.e., exact *P*-value is *P*=0.0003; *n*=6 sites).

Filopodia formation on dendrites is known to be triggered by synaptic activity, subsequent Ca^2+^ elevation and activation of Ca^2+^-dependent enzymes^27^. We therefore hypothesized that microglia contacts may trigger Ca^2+^ transients, as have been previously seen in dendrites surrounded by microglia^28^. To test this possibility, we *in utero* electroporated the genetically encoded green calcium indicator (GCaMP6m), along with tdTomato, into L2/3 neurons in Iba1-EGFP mice. Subsequent *in vivo* imaging in awake P8–P10 mice detected local neuronal Ca^2+^ responses in dendrites following microglial contacts that were frequently followed by filopodia formation (Fig. 2a–c). Such local Ca^2+^ responses were not detected at these contact sites before the microglia contact and typically disappeared following the contact. Local Ca^2+^ responses were also not observed at dendritic locations 20 μm adjacent to the contact point during the same imaging periods (Fig. 2d; 20 μm *n*=3, before contact *n*=4, during contact *n*=11 and after contact *n*=7 dendrites in 8 mice). The fliopodia formation rate was significantly higher in dendrites in which a contact-induced Ca^2+^ response was observed (Fig. 2e; 86.1±8.4% versus 16.0±7.1% in dendrites without a Ca^2+^ response; unpaired *t*-test, exact *P*-value is *P*=0.00006; exact *t*-value is *t*(7)=4.03; with Ca^2+^: *n*=7 animals, without Ca^2+^: *n*=16 animals). The lifetime of filopodia formed on dendrite in which a microglia-induced Ca^2+^ response (Fig. 2f; 20.7±2.7 min, awake mice) was longer than filopodia formed on dendrites without a Ca^2+^ response (7.1±3.6 min, awake mice) or in dendrites in anaesthetized mice (7.9±1.2 min; one-way ANOVA, *post-hoc* Bonfferoni test, exact *P*-values are *P*=0.002 (awake Ca^2+^, awake no Ca^2+^), *P*=0.0003 (awake Ca^2+^, anaesthesia), *P*=1 (awake no Ca^2+^, anaesthesia), exact F-value is F(2, 25)=12; awake Ca^2+^: *n*=7 dendrites, awake no Ca^2+^: *n*=5 dendrites, anaesthesia: *n*=16 dendrites). To further probe the signalling mechanisms linking microglia contact and dendritic filopodia formation, we transfected cortical slice cultures from Iba1-EGFP mice with lifeact-mCherry. Microglia contact was followed by accumulation of F-actin in dendrites and subsequent filopodia formation, consistent with the known role of actin in filopodia formation^29^,^30^ (Fig. 2g,h and Supplementary Movie 2). The relative fluorescent intensity at microglia contact sites (1.4±0.04) was significantly greater than the basal intensity at dendritic regions adjacent (by 10 μm) to the contact site (Fig. 2i; 1.1±0.01; paired *t*-test, exact *P*-value is *P*=0.0003; exact *t*-value is *t*(5)=8.87; *n*=6 sites). Together, this data suggests that microglia initiates filopodia formation via Ca^2+^-induced actin accumulation, with microglial contact-induced filopodia being more stable.

### Minocycline reduces the filopodia formation rate

**Figure 3:**
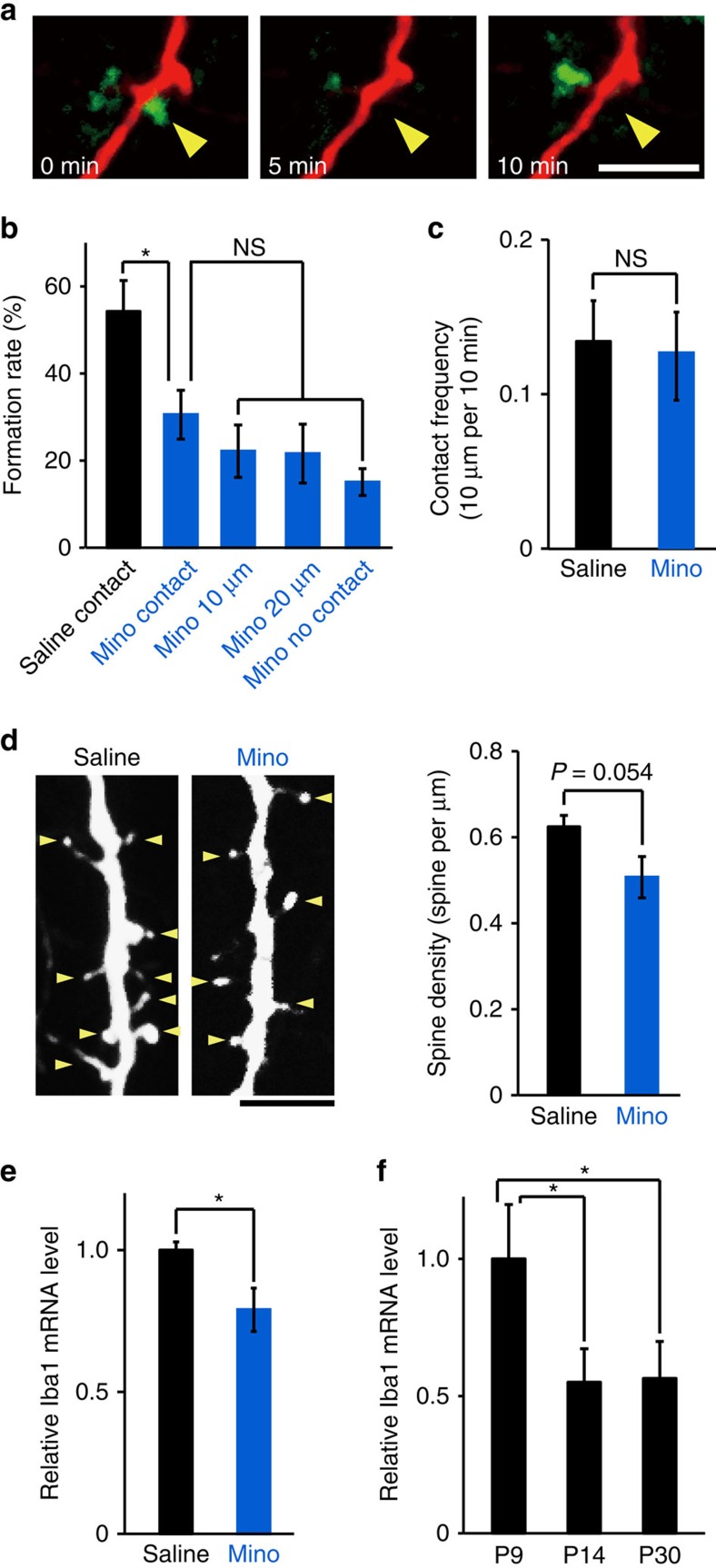
Treatment with Mino decreases filopodia formation. (**a**) Time-lapse *in vivo* images of microglia–dendrite interaction in Mino-treated mice, illustrating contact that was not followed by filopodia formation. Yellow arrowhead indicates the contact point. Scale bar, 5 μm. (**b**) Filopodia formation rate at microglia contact points (contact) was significantly decreased in Mino-treated mice, as compared with control saline-injected mice, and was the same as observed at dendritic regions 10 or 20 μm adjacent to the contact point, or across the the whole dendrite (excluding the contact region) (one-way ANOVA, *post-hoc* Tukey's test, error bars are mean ±s.e.m.; exact *P*-values are *P*=0.016 (Mino, Saline), *P*=0.96147 (Mino contact, Mino 10 μm), *P*=0.84726 (Mino contact, Mino 20 μm), *P*=0.57751 (Mino contact, Mino no contact); Saline *n*=13, Mino *n*=10 animals). (**c**) Mino did not significantly affect the microglia–dendrite contact frequency (unpaired *t*-test, error bars are mean ±s.e.m.; exact *P*-value is *P*=0.86; Saline *n*=13, Mino *n*=10 animals). (**d**) The spine density in basal dendrites of L2/3 pyramidal neurons from Mino (right)-injected mice tended to be smaller than in saline-injected mice. Left panel, sample image, yellow arrowheads indicate spines. Scale bar, 5 μm. Right panel, mean spine densities (unpaired *t*-test, error bars are mean ±s.e.m.; exact *-* value is *P*=0.054; Saline *n*=9, Mino *n*=10 mice). (**e**) Iba1 mRNA levels were higher in the cortex of P8 saline-injected mouse compared with age-matched Mino-injected mice (unpaired *t*-test, error bars are mean ±s.e.; exact *P*-value is *P*=0.019; Saline *n*=4, Mino *n*=4 animals). (**f**) Cortical Iba1 mRNA levels were significantly decreased over age, from P9 through to P14 or P30 mice (one-way ANOVA, *post-hoc* Bonfferoni test, error bars are mean ±s.e.; exact *P*-values are *P*=0.032(P9, P14), *P*=0.037(P9, P30); P9 *n*=3, P14 *n*=3, P30 *n*=3 animals).

Microglia at P8–P10 have a more activated phenotype as compared with P12–P14 mice and adult mice^31^, as we also confirmed by morphological criteria (Supplementary Fig. 2) and relative ionized Ca^2+^ binding adapter molecule 1 (Iba1) messenger RNA expression levels (Fig. 3f; one-way ANOVA, *post-hoc* Bonfferoni test, error bars are mean ±s.e.; exact *P*-values are *P*=0.032(P9, P14) *P*=0.037(P9, P30), exact F-value is F(2,6)=8.6; P9 *n*=3 animals, P14 *n*=3 animals, P30 *n*=3 animals). Daily intraperitoneal (i.p.) injections of minocycline (Mino; 75 mg kg^−1^) from P5 through to the imaging day (P8–P10) reduced activation of microglia as indicated by a decreased Iba1 mRNA expression (Fig. 3e; unpaired *t*-test, exact *P*-value is *P*=0.019, exact *t*-value is *t*(6)=2.63; saline *n*=4 animals, Mino *n*=4 animals) and significantly reduced the contact-induced filopodia formation rate, from 54±7.0% with saline treatment to 30±5.6% with Mino treatment. In Mino-treated mice, the filopodia formation rate at contact sites and non-contact sites on dendrites was not significantly different (Fig. 3a,b; one-way ANOVA, *post-hoc* Tukey's test; exact *P*-values are *P*=0.016 (Mino, Saline), *P*=0.96147 (Mino contact, Mino 10 μm), *P*=0.84726 (Mino contact, Mino 20 μm), *P*=0.57751 (Mino contact, Mino no contact), exact F-value is F(4, 44)=7.79; saline *n*=13 animals, Mino *n*=10 animals). The decrease in filopodia formation rates in Mino-treated mice was not due to a change in contact frequency (Fig. 3c; unpaired *t*-test; exact *P*-value is *P*=0.86, exact *T*-value is *t*(21)=0.18; saline *n*=13 animals, Mino *n*=10 animals). Together, this suggests that microglia activation is a crucial factor for microglia-induced filopodia formation. The mean spine density at P11 in Mino-treated mice was smaller than in saline-treated control mice (Fig. 3d), but this was not significantly different (unpaired *t*-test; exact *P*-value is *P*=0.054; exact *T*-value is *T*(17)=2.07; saline *n*=9 mice, Mino *n*=10 mice).

### Microglia ablation reduces the number of functional synapses

**Figure 4:**
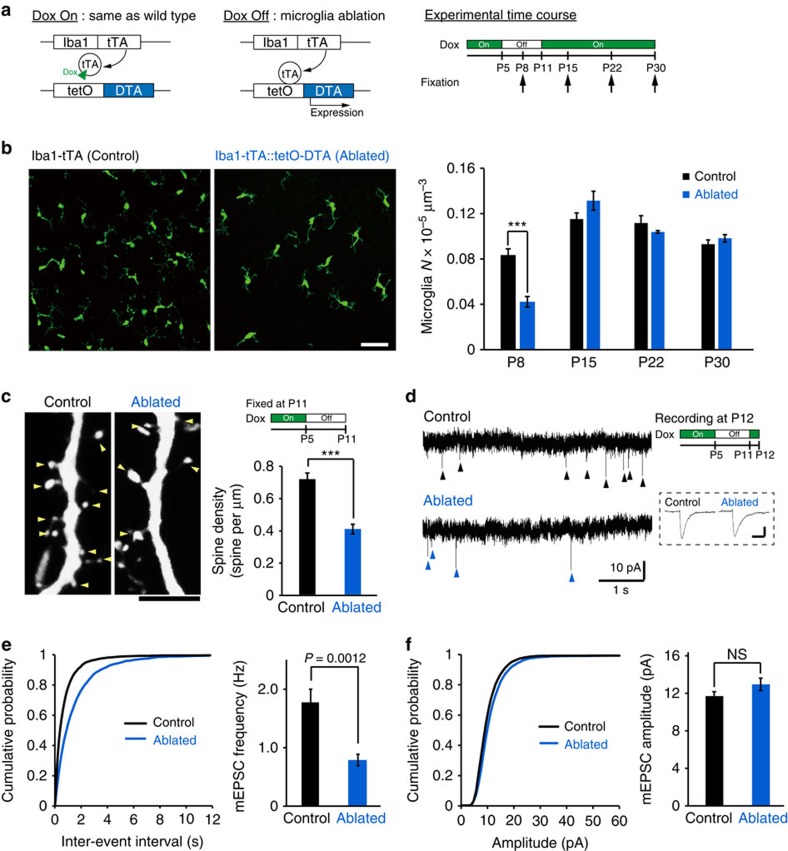
Microglia ablation reduces the number of functional synapses. (**a**) Left panel: schematic of the ablation strategy used to reduce microglia by transient withdrawal of dietary Dox. Arrows in the right panel indicate brain fixation times for microglial histology. (**b**) Sample fluorescent images from P8 mice (left) and averaged data (right) showing reduced Iba1 (microglia) immune reactivity in P8 Iba1-tTA::tetO-DTA transgenic mice following Dox withdrawal from P5 to P10, as compared with the Iba1-tTA mice (control) at P8. Scale bar in left panel, 50 μm. The recovery of microglial density by P15 (right panel) is noteworthy (unpaired *t*-test, error bars are mean ±s.e.m.; P8: exact *P*-value is *P*=9.3E−5, P15: exact *P*-value is *P*=0.15, P22, exact *P*-value is *P*=0.27, P30: exact *P*-value is *P*=0.33; P8: (control *n*=6, ablated *n*=8 animals), P15: (control *n*=4, ablated *n*=4 animals), P22: (control *n*=4, ablated *n*=4 animals), P30: (control *n*=4, ablated *n*=4 animals)). (**c**) Typical images of dendritic spines (yellow arrowheads) in L2/3 pyramidal neurons from control (left) and microglia-ablated mice (right). Scale bar, 5 μm. The mean spine density (right panel) was significantly reduced in microglia-ablated mice (unpaired *t*-test, error bars are mean ±s.e.m.; exact *P*-value is *P*=6.26E−5; Control *n*=7, Ablated *n*=6 animals). (**d**) Representative current traces from voltage-clamped L2/3 pyramidal neurons from P12 control and microglia-ablated mice, 6 days after Dox withdrawal, as indicated by the upper right experimental schematic. Arrowheads indicate mEPSCs. Inset indicates averaged (*n*∼10) mEPSCs from each condition. Scale bar, 5 pA, 10 ms. (**e**) Typical cumulative mEPSC frequency (**e**, left) and amplitude (**f**, left) distributions from a control and microglia-ablated mouse. Accompanying bar graphs show averaged date for mEPSC frequency (**e**, right) and mEPSC amplitude (**f**, right). Mean frequency was significantly reduced by transient microglia ablation (unpaired *t*-test, error bars are mean ±s.e.; exact *P*-values are *P*=0.0012 (**e**, right), *P*=0.14 (**f**, right); Control *n*=10 neurons from 5 animals, Ablated *n*=9 neurons from 3 animals).

To further evaluate the functional consequences of microglia on spinogenesis, we used a genetic strategy to ablate microglia *in vivo* (Fig. 4a). We cross-bred Iba1-tetracycline transactivator (Iba1-tTA) mice with tetracycline operator-diphtheria toxin A (tetO-DTA) mice. Transiently withdrawing doxycycline (Dox) from the maternal diet between P5 and P11 reduced the number of microglia at P8 by ∼50%, with microglial numbers recovering to control levels by P15 (Fig. 4b, unpaired *t*-test, P8: exact *P*-value is *P*=9.3E−5, exact *t*-value is *t*(12)=−5.7; P15: exact *P*-value is *P*=0.15, exact *t*-value is *t*(6)=1.64; P22: exact *P*-value is *P*=0.27, exact *t*-value is *t*(6)=−1.2; and P30: exact *P*-value is *P*=0.33, exact *t*-value is *t*(6)=1.06; P8: (control *n*=6 animals, ablated *n*=8 animals), P15: (control *n*=4 animals, ablated *n*=4 animals), P22: (control *n*=4 animals, ablated *n*=4 animals) and P30: (control *n*=4 animals, ablated *n*=4 animals)). This transient partial ablation of microglia reduced the density of spines when we quantified this in P11 mice (from 0.72±0.04 to 0.41±0.03 spines per μm; Fig. 4c; unpaired *t*-test, error bars are mean ±s.e.m.; exact *P*-value is *P*=6.26E−5, exact *t*-value is *t*(11)=6.2; control *n*=7 animals, ablated *n*=6 animals). DTA expression in microglia does not alter cytokine levels, apoptosis nor pre-synapse number^22^. The reduction of the spine density in microglia-ablated mice was also associated with a significant and selective decrease in the frequency of miniature excitatory postsynaptic currents (mEPSCs) in L2/3 neurons in brain slices isolated from P12 mice, from 1.7±0.2 to 0.8±0.1 Hz (Fig. 4d,e; unpaired *t*-test, exact *P*-value is *P*=0.0012, exact *t*-values are *t*(17)=−3.88(F); control *n*=10 neurons from 5 animals, ablated *n*=9 neurons from 3 animals). The distribution of mEPSC amplitudes was not affected by microglia ablation (Fig. 4d,f; unpaired *t*-test; exact *P*-value is *P*=0.14, exact *T*-value is *t*(17)=1.56). This data demonstrates that microglia ablation during the specific period of microglia-induced filopodia formation reduced the subsequent number of functional synapses.

### Microglia ablation selectively reduces synapses from L4

**Figure 5:**
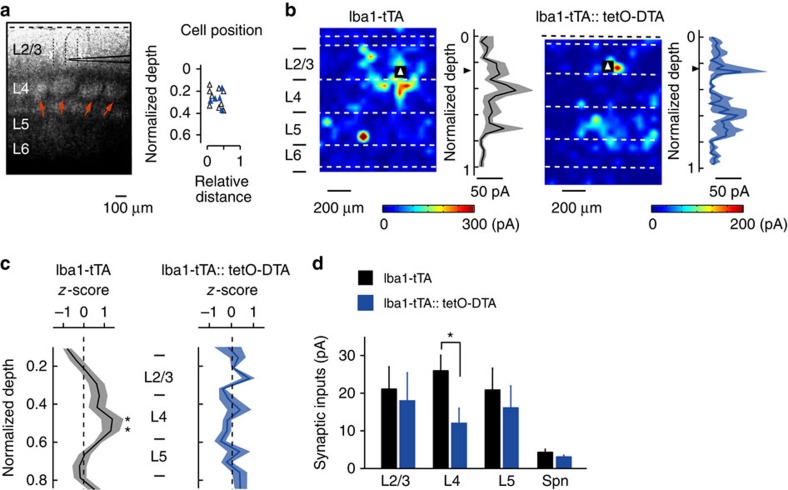
Microglia ablation selectively impairs L4 to L2/3 synaptic connections. (**a**, left) Differential interference contrast image of a slice of barrel cortex, showing the cell layers and with a recording electrode positioned in L2/3. Red arrows indicate L4 barrels. Scale bar, 100 μm. (**a**, right) Scatterplot of the position of L2/3 pyramidal cells from which recordings were made, at a depth relative to layer 1 (dashed line in **a**, left) and at a horizontal distance from the centre of a single barrel (open triangles for control mice, blue triangles for DTA mice). (**b**) Representative heat maps and input strength-depth profiles (black line and grey shading represent mean and s.e., respectively) from a control (**b**, left) and microglia ablated (**b**, right) cortical slice. Photo-stimulation was applied throughout the slice with the colour and contours representing the sum the amplitudes of EPSCs evoked by stimulation at each position. Triangles indicate the location of the recorded L2/3 pyramidal cell. Dashed white lines indicate the laminar borders. (**c**) Averaged amplitude of excitatory synaptic currents in response to stimulation from the top of L2/3 through to L6. Black line and grey or blue shadow indicate mean and s.e., respectively (Control versus DTA mice for stimulation of each location) (unpaired *t*-test; exact *P*-value is *P*=0.031 or *P*=0.044; Control *n*=9 from 4 animals, DTA *n*=5 from 3 animals). (**d**) The strength of synaptic inputs (mean±s.e.m.) grouped across the whole layer and spontaneous EPSCs (Spn) (unpaired *t*-test; exact *P*-value is *P*=0.032).

In a final set of experiments, we probed the possible functional consequences of microglia contact-induced filopodia formation, hypothesizing that filopodia formed by microglia may predominantly contribute to the formation of specific connections during this developmental period. To test this hypothesis, we recorded monosynaptic EPSCs from L2/3 pyramidal cells above a cortical barrel using whole-cell patch clamp recordings in brain slices from control (Iba1-tTA) and microglia ablated (Iba1-tTA::tetO-DTA) P23–P25 mice, by which time barrel cortical circuits have matured. EPSCs were evoked by glutamate uncaging with laser scanning photostimulation across L2–L5 (Fig. 5a). Such photostimulation-evoked EPSCs are largely due to single action potentials in individual presynaptic neurons^32^ and thus can be used to quantify synaptic inputs originating from the different cortical layer neurons. In both control and microglia ablated mouse slices, L2/3 neurons received inputs from L2–L5. In control mice, L2/3 neurons received stronger inputs from L4 compared with other layers, consistent with previous data^33^ (Fig. 5b,c; unpaired *t*-test between corresponding locations in control and DTA mice; exact *P*-value is *P*=0.031 or *P*=0.044; control *n*=9 from 4 animals, DTA *n*=5 from 3 animals). However, in slices from microglia-ablated DTA mice, the largest synaptic response did not arise from L4, resulting in an averaged spatial distribution of synaptic inputs that was relatively uniform across the cortical layers (Fig. 5b,c). The averaged strength of synaptic inputs originating from L4 was significantly lower in DTA mice, compared with control mice, whereas inputs from L2/3 or L5 were not significantly different (Fig. 5d; versus control rats; unpaired *t*-test; exact *P*-value is *P*=0.032). The averaged amplitude of spontaneous synaptic currents was also not different in DTA and control mice. These results indicate that microglia ablation selectively reduced excitatory synaptic connections from L4 to L2/3, suggesting that microglia-induced filopodia formation specifically promotes the establishment of feedforward synaptic connections from L4 to L2/3.

## Discussion

**Figure 6:**
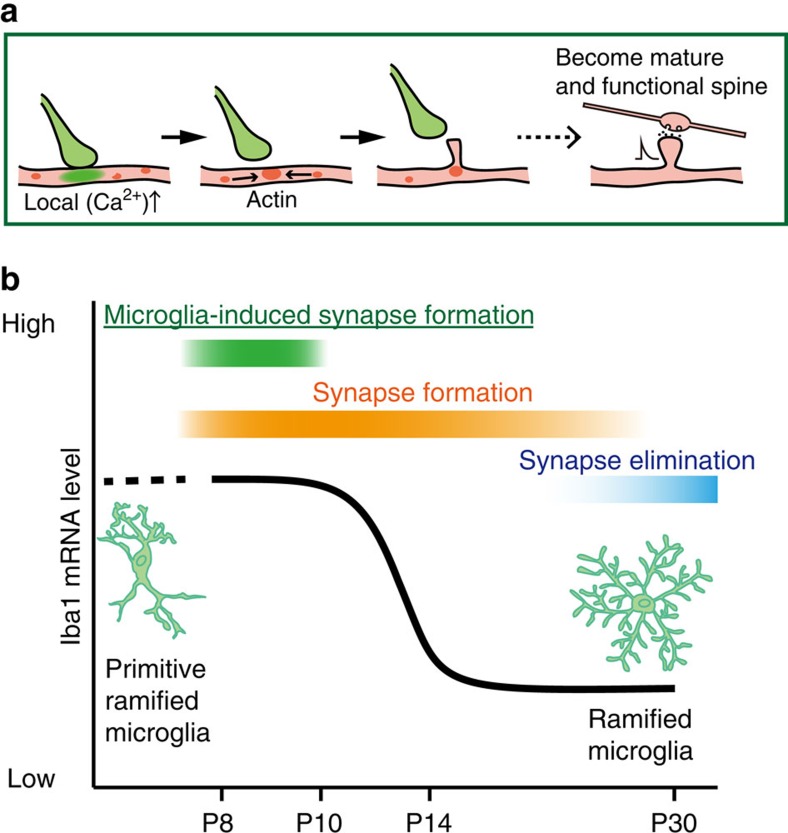
Scheme of microglia functions during synaptogenesis. (**a**) Sequence of proposed cellular events during synapse formation: Microglial contact initiates a rise in local [Ca^2+^]_i_ resulting in actin accumulation and filopodia formation. Some filopodia find presynaptic partners and mature into functional synapses. (**b**) Schematic graph depicting a change in microglia phenotype and function, from immature or activated microglia inducing filopodia formation and enhancing specific circuit synapse formation during early synaptogenesis, followed by a putative role in synapse elimination by more mature, quiescent microglia in the latter period of circuit formation.

Establishing appropriate synaptic connections during development is generally achieved by the balance between synaptogenesis and experience-dependent synapse pruning and maturation. In the mouse somatosensory cortex, an intense period of synaptogenesis (with a rapid ≈7-fold increase in synapses) begins around P8, with synapse numbers peaking around P32, before declining (by≈20%) to adult levels^26^. Synapses develop from filopodia, labile, postsynaptic protrusions that may stabilize and develop into mature spines^22^,^25^,^34^,^35^ when they connect onto presynaptic terminals. Microglia contribute to a number of aspects of neural circuit development, including to help define the neuronal population via regulation of apoptosis and neurogenesis and by phagocytosing excess synapses during synaptic pruning^11^,^15^,^17^. Our studies demonstrate a new physiological role for microglia in promoting synapses during a transient period of brain development, as contacts between microglial processes and dendritic shafts could trigger filopodia formation (Fig. 6a). Consistent with known mechanisms of filopodia formation^34^,^36^, microglial contacts could also trigger localized Ca^2+^ transients and recruitment of actin. Although filopodia formation also occurred in the absence of microglia contact, the filopodia formation rate at microglia contact sites on a dendrite was greater than that observed at non-contact sites. At least some of these microglia-induced filopodia appeared to develop into functional excitatory synapses, as ablating microglia reduced the subsequent functional spine density and frequency of excitatory synaptic transmission. Microglia may potentially be attracted to dendritic locations where the early stages of filopodia formation are already in place, or may initiate *de novo* filopodia formation. The sequence of events where Ca^2+^ transients only occur during contacts and are directly followed by actin accumulation and filopodia formation seem to favour the latter hypothesis. Some of the filopodia induced by microglia exhibited an increased stability, which may also contribute to the microglia-dependent increase in mature spines and functional synapses, and this was particularly evident in awake mice when the microglia–dendrite contact was followed by a localized Ca^2+^ transient. Interestingly, the lifetime of microglia-induced filopodia in anaesthetized mice was similar to the lifetime of non-contact filopodia. Anaesthetics are known to affect filopodia formation and stability^36^, and may have occluded potential differences in filopodia lifetime in this set of experiments.

A striking observation in our study was the transient nature of the microglia-induced filopodia formation. During early development, microglia populate the brain in an activated, amoeboid phenotype before adopting the more mature ramified surveying morphology^31^,^37^. Microglia proliferate into the barrel cortex from P5 in the amoeboid state and gradually change their phenotype to a more ramified state^38^. We similarly observed a morphological change between P8 and adults, as well as clear transitions in both cell soma size and Iba1 mRNA expression patterns between P8–P10 and P12–P14 microglia. The microglia contact-induced filopodia formation was only observed in P8–P10 mice, corresponding to the amoeboid, activated state. Consistently, inhibition of microglial activation with Mino abolished microglia-induced filopodia formation. The effects of Mino may be mediated through a change in activation phenotype, which could also include a decrease in the release of various soluble factors such as IL-10, tumour necrosis factor-α or BDNF. Activated microglia directly opposed to, or in contact with, dendrites may physically or via surface receptors promote functional spine formation. Regardless, this novel facet of microglia-induced neuronal plasticity we observe here is restricted to the activated, amoeboid phenotype. Phagocytosis of surplus synapses in developing lateral geniculate circuits also occurs early in development (around the first postnatal week) by the amoeboid microglia phenotype^17^. Experience-dependent pruning of synapses in visual cortex is evident at 4 weeks postnatal, when microglia have a mature ramified phenotype^39^. Microglial BDNF-dependent spine formation following motor learning occurs in the healthy adult rodent motor cortex when microglia are also of the quiescent, ramified morphology. Clearly, the precise functional consequences of microglia–neuron contacts on circuit plasticity—and whether this tends towards synapse elimination, synapse surveillance or spine formation—are likely to depend on the particular brain region and the specific activity or developmental pattern of synaptic plasticity occurring at that time. Our data emphasize the repertoire of actions microglia can participate in across the developmental time scales.

The different synaptic inputs to the cortex arrive during different developmental time windows and display corresponding different critical periods of activity or experience-dependent synaptic plasticity^40^. Thalamic inputs to L4/L5 neurons arise initially into the somatosensory barrel cortex (around P0) and establish the columnar or barrel organization. Local cortical inputs to L2/3 neurons subsequently arise from recurrent connections from other L2/3 neurons and from feedforward projections from L4 neurons. Although there is some overlap, the L4 connections and the corresponding critical periods of plasticity occur earlier (around P10–P14) than those from L2/3 projections (P13–P16)^40^. By extension, filopodia that appear around P8–P10 may preferentially mature into L4-derived synapses, whereas filopodia at later stages will preferentially mature into L2/3-derived synapses. Consistent with this proposal, genetic and transient ablation of approximately half the microglial population around P8 to inhibit microglia-induced filopodia formation selectively weakened synaptic inputs to L2/3 neurons from L4 stimulation. It appears that the cortical circuits use the capacity of amoeboid microglia at this specific developmental stage to increase particular synapses being formed at that time (Fig. 6b). Additional cellular mechanisms may exist to facilitate synapse formation from other inputs at other specific developmental time points.

It is unclear at this stage which signalling mechanisms may trigger the spatially restricted increase in filopodia formation rate induced by microglial contact. Numerous mechanisms and molecules have been identified to promote spine formation^41^. For example, microglia may increase neuronal activity and/or NMDA (*N*-methyl-D-aspartate)-mediated Ca^2+^ transients by directly releasing glutamate or via enhanced release of glutamate from approaching presynaptic neurons^24^. Receptor-mediated direct physical coupling between microglia and dendrites may be involved, similar to that proposed for synapse elimination. In the developing lateral geniculate nucleus, microglia phagocytosis of synapses involves interactions between immune complement receptor molecules C3 and C3R^17^, whose expression depends on neuronal activity. In the hippocampus, fractalkine receptor–ligand interactions contributed to synapse phagocytosis^16^. Other complementary molecules such as telencephalin, nectin, IL-1 receptor accessory protein-like 1 and C1q family have been reported to be involved in synapse formation^42^,^43^,^44^,^45^,^46^. Neural cell adhesion molecule (NCAM2) is expressed in both neurons and microglia, and could trigger intracellular Ca^2+^ elevation that may subsequently recruit actin and elicit filopodia formation^47^,^48^. Microglia secrete a variety of soluble factors, including cytokines, neurotrophic factors and neurotransmitters, with this pattern changing dependent on different phenotypes from quiescent to challenged^7^. For motor learning-induced spine formation, release of BDNF by microglia is critical^22^, whereas microglial release of IL-10 enhanced spine formation in cultured neurons^23^. Microglia can also release tumour necrosis factor-α, which can signal via various receptors and pathways to increase expression of N-cadherin to increase spine density^49^. Our characterization of the precise time course and transient nature of microglia-induced filopodia formation should assist experiments aimed at identifying the underlying signalling pathways.

A number of psychiatric brain disorders, such as schizophrenia and autism, involve disruptions in synapse number, morphology or function, with a pathogenesis that is believed to initiate with synapse development^50^. In autism spectrum disorders in particular, there is a surplus of spines in the cortex during early development in both human and mouse models, and dysfunction in the turnover rates of cortical spines is a common feature in a number of mouse models of autism^51^. Given the association between maternal infection and autism and schizophrenia incidence, microglia are possible pathogenic candidates. Our studies here provide an additional aspect of microglia–neuron interactions—developmental spine formation and specific neuronal circuit connections—and further investigations into the underlying mechanisms of microglia–neuron interactions may shed light on the pathophysiology of brain diseases and provide potential strategies for restoring synapse function.

## Methods

All animal experiments were approved by the Animal Research Committee of the National Institutes of Natural Sciences.

### Animals and microglia inhibition

To visualize microglia, we used the Iba1-EGFP transgenic mouse, which expresses EGFP under the control of the Iba1 promoter, which is specific for microglia and macrophages^52^. For microglia ablation experiments, double transgenic mice were generated by crossing Iba1-tTA mice^53^ and tetO-DTA mice^54^. Withdrawal of Dox in the feed of these mice leads to selective expression of the DTA in microglia. All transgenic mice were derived from the C57BL/6J strain. Transgene induction was inhibited by rearing mice with standard chow containing Dox 0.1 g kg^−1^. For transgene induction, Dox-laced chow was replaced with Dox-free standard chow from P5 through to P11 (for analysis of spine density). Both male and female mice were used for all experiments. Mice (single mothers and their litter) were housed on a 12-h light/dark cycle. Mino hydrochloride (M9511-1G, Sigma-Aldrich, Tokyo, Japan) was used to pharmacologically inhibit microglia and injected daily (i.p., 75 mg kg^−1^) from P5 through to the experimental day (P8–P10, for *in vivo* imaging of microglia–dendrite contacts; P11 for analysis of spine density). Control mice received i.p. saline injections with the same dosing schedule.

### *In utero* electroporation to visualize neurons

To visualize L2/3 pyramidal neurons, we performed *in utero* electroporation of embryos at embryonic days (E) 14 or E15 (ref. 55). At this age, intraventricular injection and electroporation only transfects neurons migrating into the cortex. Under anaesthesia (1.7% isoflurane), the uterus was exposed and ∼1.5 μl of plasmid solution was injected into the lateral ventricle of each embryo using a glass pipette (tip diameter: 50–100 μm). The head of a single embryo was then placed between tweezer-type electrodes with 5 mm tip diameters (CUY650-P5; NEPA Gene, Chiba, Japan) and square electric pulses (35 V; 50 ms) were applied to the electrodes 5 times, at 950 ms intervals, using an electroporator (CUY21E; NEPA Gene). Each embryo was quickly returned to the abdomen following the electrical stimulus and once all embryos were electroporated the peritoneal membrane was sutured back together. The mother's skin was bound by clips (12032-07, Muromachi Kikai, Tokyo, Japan) to avoid reopening the surgical wound.

We used a plasmid, which contained floxed RFP (pCALNL-tdTomato; at 0.4 μg μl^−1^), for dendritic spine imaging, and floxed green calcium indicator (pCALNL-GCaMP6m; at 0.8 μg μl^−1^) for dendritic calcium imaging, which was co-injected with a plasmid carrying Cre recombinase (pCAG-Cre (Addgene), 0.01 μg μl^−1^). To evaluate whether the injection location was successful, Fast Green FCF 0.25 μg μl^−1^ (F7258-25G, Sigma-Aldrich) was included in the plasmid solution.

### *In vivo* two-photon imaging

Electroporated Iba1-EGFP mice (P8-P10) were anaesthetized with urethane (1.7 g per kg body weight, i.p. and atropine, 0.4 mg kg^−1^, i.p.). Surgery and imaging were performed on a warming plate. After removal of the scalp, a cranial window (1.6 mm in diameter) was made over the primary somatosensory barrel cortex (1 mm posterior from bregma and 2.5 mm lateral from the midline). A cover glass was placed over the cranial window and fixed with adhesive glue (Aron Alpha, Konishi, Osaka, Japan) and dental cement (Quick Resin, SHOFU, Kyoto, Japan). A custom-made imaging chamber surrounded the cranial window and was perfused with warm water (32–34 °C) during imaging.

Two-photon imaging was performed with a Ti:sapphire laser (Mai Tai HP, Spectra-Physics, Tokyo, Japan) operating at 960 nm wavelength. A laser scanning system (Olympus FLUOVIEW, Olympus, Tokyo, Japan) and an upright microscope (BX61WI, Olympus) with a water-immersion objective ( × 25, 1.05 numerical aperture (NA); Olympus) was used for image acquisition. Fluorescence was separated by a 570 nm dichroic mirror with 495–550 nm (green channel: for EGFP fluorescence detection) and 570–630 nm (red channel: for DsRed-express or tdTomato fluorescence detection) emission filters, and detected by photomultipliers. For time-lapse imaging, *Z*-stack images (512 × 512 pixels, 0.099 μm per pixel, 0.5 μm *Z*-steps) were taken every 5 min for between 30 min and 2 h at a depth of 45–250 μm from the cortical surface. For short interval imaging, XYt images were taken once every 1.6 s, or once per minute, for 27 min.

For dendritic Ca^2+^ imaging in awake mice, cranial window (1.5 mm) surgery was performed under isoflurane anaesthesia (1.5% isoflurane in pure O_2_). After an hour recovery, calcium imaging was performed in the awake state. XYt images were taken at 8 Hz for 30 min (256 × 128 pixels, 0.25 μm per pixel). Two-photon imaging was performed with a Ti:sapphire laser (Mai Tai HP, Spectra-Physics) operating at 940 nm wavelength. To detect calcium transients, we defined a small region of interest (ROI) (2 × 2 μm) along the dendritic shaft and calculated an average basal Ca^2+^ fluorescence. A significant Ca^2+^ elevation response was defined if the peak amplitude of Δ*F*/*F* was greater than three times the s.d. of the basal Ca^2+^ level. A localized Ca^2+^ response was defined as one that was restricted to just a small portion of the dendrite (<12 μm), in contrast to transients associated with back-propagating action potentials.

### Analysis of *in vivo* microglia and neuron interactions

Image stacks were visually inspected in ImageJ (National Institutes of Health, USA), to determine regions of co-localization of GFP-labelled microglia and RFP-labelled dendrites, and then subsequently further examined in greater detail across all image time points and *Z*-sections using ImageJ, to confirm real contacts. To reduce noise, images were filtered with a 3 × 3 pixel median filter after background subtraction. Contacts were determined by overlap between the red (dendrite) and the green (microglia) channel, after independently determining the baseline thresholds for each channel independently. Identified overlaps were defined as contacts only if the red and the green channel had overlapping pixels in at least two *Z*-sections and if the overlap extended beyond twice the spatial resolution.

Dendrites that retracted during time-lapse imaging were excluded from analysis. Dendritic spines or filopodia were identified as protrusions that were >0.4 μm from the dendrite border. A spine was defined as a protrusion with a head structure, whereas filopodia lacked head structures. This categorization included stubby structures as filopodia. This is appropriate as these types of ‘spines' are unstable during development and frequently disappear^56^. Contact-induced filopodia formation rates were defined as the incidence in which a filopodia was formed at a microglia contact region. The contact region on a dendrite was defined as a 2 μm zone (±1 μm from the estimated centre of the microglia–dendrite contact) and a protrusions had to appear within 10 min of the onset of the microglia–dendrite contact to be counted as being formed by microglia (it is noteworthy that Fig. 1c includes all protrusions that appeared within 20 min after microglia contact for comparison). Between 1 and 27 microglia–dendrite contacts were observed in different dendrites in each animal and the incidence of a contact-induced filopodia were averaged to give a single formation rate for each animal. The formation rate of protrusions at dendritic regions 10 μm (±1 μm) and 20 μm (±1 μm) adjacent to the microglia contact region were calculated as a measure of the control (or putative non-contact) filopodia formation rates. This analysis counted the incidence of a protrusion forming at these non-contact regions along the same dendrites at any time during the same imaging period over which contact-induced formation rates were measured. To quantify the formation rate across a single dendrite, the number of any protrusions appearing along the whole region of a dendrite during the imaging was counted (excluding those at the contact region) and normalized by the dendritic length. Dendrite length was expressed in multiples of 2 μm blocks (for example, 10 μm=5), to compare with the 2 μm length unit used to define contact and non-contact regions. It should be noted that formation ‘rate' as used in the study is related to the incidence of filopodia formation per unit length, rather than over a given time period.

### Brain fixation and immunohistochemistry

To quantify microglia morphology (Iba1-EGFP mice) and spine density (electroporated Iba1-tTA::tetO-DTA or Iba1-tTA, or wild-type C57BL/6J mice), mice were deeply anaesthetized with ketamine (0.13 mg g^−1^, i.p.) and xylazine (0.01 mg g^−1^, i.p.), and transcardially perfused with 4% paraformaldehyde. The brain was dissected out, postfixed for 2 days in 4% paraformaldehyde at 4 °C and 100 μm thick coronal slices that included the barrel cortex were sectioned with a vibratome (VT1000S; Leica, Tokyo, Japan). Sections were mounted with VECTASHIELD mounting medium (H-100, Funakoshi, Tokyo, Japan) and imaging was performed within 7 days of sectioning.

Thirty-micrometre-thick coronal sections containing barrel cortex were similarly cut for immunohistochemistry. Slices were incubated in blocking solution (0.1% normal goat serum, 0.05% NaN_3_, 0.5% Triton X-100 in 0.1 M PBS) for 30 min. After washing with 0.1 M PBS, sections were incubated overnight at 4 °C with primary antibodies (1:500 dilution; anti-Iba1 antibody, 019-19741, Wako, Osaka, Japan)^6^. Slices were incubated overnight at 4 °C with secondary antibodies (1:300 dilution; Alexa Fluor 633 Goat Anti-Rabbit, Life Technologies, Carlsbad, CA, USA). Finally, sections were mounted with VECTASHIELD mounting medium with 4,6-diamidino-2-phenylindole (H-1200, Funakoshi) and imaging was performed within 7 days.

### Image analysis for microglia morphology

Iba1-EGFP mice were used to visualize microglia, using thick (100 μm) brain slices (as described above) to enable measurement of the complete extent of microglial processes. Confocal imaging was performed with a multi argon laser operating at a wavelength of 488 nm. A laser scanning system (Nikon A1, Nikon, Tokyo, Japan) and an inverted microscope with water-immersion objective ( × 40, 0.95 NA, Nikon) was used for image acquisition. Fluorescence was separated by a dichroic mirror with 495–550 nm (green channel: for EGFP fluorescence detection) emission filters and detected by photomultipliers. *Z*-stack images (512 × 512 pixels, 0.198 μm per pixel, 0.5 μm *Z*-step) were used for morphological analysis.

Image processing was performed using ImageJ software. Maximum intensity projections of *Z*-series stacks were created. The extent of microglia process ramification was quantified by measuring the area circumscribed by the distal ends of microglial processes using the segmented line tool in ImageJ. Microglia size was quantified by circumscribing the cell body area using an intensity threshold tool in MetaMorph (Molecular Devices, Tokyo, Japan). The number of microglia primary processes was manually counted, with primary processes defined as those starting from the microglia soma.

### Image analysis for spine density

*In utero* electroporated mice were used for visualization of L2/3 pyramidal neuron dendritic spines. Brains were fixed and sectioned (100 μm) as described above. Confocal imaging was performed with 10 mW 561 nm solid laser. A laser scanning system (Nikon A1, Nikon) and an inverted microscope with a water-immersion objective ( × 60, 1.2 NA, Nikon) was used for image acquisition. Fluorescence was again separated by a dichroic mirror with 572–700 nm emission filter (red channel: for tdTomato fluorescence detection) and detected by photomultipliers. *Z*-stack images (512 × 512 pixels, 0.08 μm per pixel, 0.5 μm *Z*-step) were used to calculate spine density.

Dendritic spines were identified in a series of *Z*-stack images and counted using ImageJ software. Serial stack images were used to assist in the delineation of individual spines in dendritic regions where spine density was high. Serial *Z*-stacks of different optical sections were used to identify individual spines with greater certainty. For this analysis, all dendritic protrusions with a clearly recognizable stalk were counted as spines. Spine number was divided by the length of the dendritic segment to generate dendritic spine density, expressed as number per micrometre.

### Slice culture and biolistic transfection

Cortical slice cultures were derived from 350 μm thick slices from P5 Iba1-EGFP mice and used to examine actin dynamics following microglia contact. After 1 day *in vitro*, cortical pyramidal neurons were transfected with a ballistic gene transfer gun using gold beads (4–6 mg) coated with 10 μg of the pCMV-lifeact-mCherry plasmid. At 4 days *in vitro*, slice cultures were mounted on a microscope stage for imaging and perfused with warmed (32–34 °C) artificial cerebrospinal fluid (ACSF), containing 126 mM NaCl, 2 mM KCl, 2 mM CaCl_2_, 24 mM NaHCO_3_, 1.2 mM NaH_2_PO_4_, 1.3 mM MgSO_4_ and 10 mM glucose. Two-photon imaging was used, with a 960 nm excitation wavelength, as described above for *in vivo* imaging. A water-immersion objective ( × 25, 1.05 NA, Olympus) was used for image acquisition. Fluorescence was separated by a 570 nm dichroic mirror with 495–550 nm (green channel: for EGFP/microglia fluorescence detection) and 570–630 nm (red channel: for mCherry/actin in neuronal dendrites fluorescence detection) emission filters, and detected by photomultipliers. Actin accumulation images were acquired using real-time imaging at a single XY image plane acquired every 1.6 or 5.0 s for 27 or 30 min, respectively. Images were analysed using ImageJ. Microglia contact was defined as overlap of green fluorescence intensity within a (red) dendritic region, whereas segregation of lifeact-mCherry was defined as an increase of red fluorescence intensity within the microglia contact region in the dendrite. Filopodia formation was defined as the red fluorescence intensity protruding from pre-contact border of the dendrite. The background intensity of the image regions of interest in the absence of neuronal or microglia structures was subtracted from the intensity of the microglia and filopodia regions. The intensity of an adjacent region of the same dendrite, but without microglia contact or the presence of a filopodia or spines, was used as background for the dendritic aggregation of the lifeact-mCherry fluorescence signal. Fluorescence values were normalized to the maximum intensity of each signal. Fluorescent intensity was normalized using a value of 25% of the maximum intensity as baseline (*F*_0_). To quantify contact induced actin aggregation, the relative intensity (*F*/*F*_0_) was averaged between 5–10 min after the onset of microglia–dendrite contact and the *F*/*F*_0_ at the contact region compared with that at a 10 μm adjacent region

### Quantitative reverse transcriptase–PCR for mRNA analysis

Mice were deeply anaesthetized with ketamine (0.13 mg g^−1^, i.p.) and xylazine (0.01 mg g^−1^, i.p.), transcardially perfused with 0.1 M PBS, which contained 5 mM EDTA and brains were then dissected. Total RNA was extracted from neocortical tissue samples using RNeasy Plus Mini Kit (74134, Qiagen), then reverse transcription was performed by Transcriptor First Strand cDNA Synthesis Kit (04896866001, Roche). Real-time PCR was performed using FastStart Essential DNA Green Master (06402712001, Roche Applied Science) on LightCycler 96 (05815916001, Roche Applied Science) with Iba1 exon primers (5′-TCCCAAATACAGCAATGATGAG-3′, 5′-GCATTCGCTTCAAGGACATAAT-3′) and GAPDH primers (5′-AATGCATCCTGCACCACCAAC-3′, 5′-TGGATGCAGGGATGATGTTCTG-3′). Fold change was calculated using the ΔΔCt method and normalized by the values obtained with saline-injected mice or P9 mice.

### Measurements of spontaneous synaptic currents

Acute brain slices were prepared from Iba1-tTA::tetO-DTA or Iba1-tTA mice at P12 following anaesthesia with ketamine (0.13 mg g^−1^, i.p.) and xylazine (0.01 mg g^−1^, i.p.), and transcardial perfusion with oxygenated (95%O_2_/5%CO_2_) slicing solution containing (in mM): 230 sucrose, 26 NaHCO_3_, 2 KCl, 1 MgCl_2_, 1 KH_2_PO_4_, 0.5 CaCl_2_ and 10 glucose. Mice were decapitated and the brains were rapidly removed, and 350 μm thick coronal cortical slices were cut in cold slicing solution. Slices were stored in oxygenated ACSF (as described above for slice culture imaging) at 34 °C for at least 45 min before being transferred to the recording chamber on the stage of an upright microscope and viewed with a × 40 water-immersion objective. Slices were continuously perfused with oxygenated recording ACSF and recordings were obtained at room temperature (around 25 °C). Whole-cell voltage-clamp recordings (at a holding potential of −70 mV) were made from the somata of visually identified barrel cortex L2/3 pyramidal neurons. Patch pipettes (5–8 MΩ) were constructed from borosilicate glass capillaries and filled with an internal solution containing (mM): 9 CsCl, 130 CH_3_SO_3_Cs, 2 EGTA, 10 HEPES, 4 Mg-ATP and 0.4 Na-GTP, pH adjusted to 7.3 with Tris. To isolate mEPSCs, 0.3 μM tetrodotoxin and 10 μM SR95531 were continuously perfused once a stable recording was obtained. Only cells with *R*_series_≦25 MΩ and *R*_input_≧200 MΩ were included for analysis. No corrections for liquid junction potentials were applied and no series resistance compensation was used.

### Laser uncaging photostimulation evoked excitatory postsynaptic currents

Coronal slices of barrel cortex (300 μm thick) were prepared from P23–P25 mice under deep anaesthesia with isoflurane and kept in normal artificial cerebrospinal fluid (ACSF) containing (in mM): 126 NaCl, 3 KCl, 1.3 MgSO_4_, 2.4 CaCl_2_, 1.2 NaH_2_PO_4_, 26 NaHCO_3_ and 10 glucose at 33 °C, as described previously^32^. For whole-cell recording, patch pipettes (4–6 MΩ) were filled with a solution containing (in mM): 130 K-gluconate, 8 KCl, 1 MgCl_2_, 0.6 EGTA, 10 HEPES, 3 MgATP, 0.5 Na_2_GTP and 10 Na-phosphocreatine (pH 7.3 with KOH). For analysis, we selected cells with a high seal resistance (>1 GΩ) and a series resistance <25 MΩ. The recording of photostimulation-evoked EPSCs and analysis of the EPSCs was conducted as described previously^57^. Photostimulation was achieved by focal photolysis of Rubi-caged glutamate with 5 ms flashes of blue light (440 nm) from a diode laser. The light was focused on the slices through a × 4, 0.16 NA microscope objective. Laser power was set to 5 mW at the specimen plane. This resulted in the generation of action potentials in neurons with cell bodies mostly within ∼100 μm of the centre of the illuminated spot. Photostimulation-evoked EPSCs were recorded from L2/3 pyramidal neurons within a single L4 barrel. Usually, the photostimulations were applied once every 5 s to each of 16 × 20 sites surrounding the recorded cells in a quasi-random sequence. The maps of photostimulation sites were aligned to laminar borders in fixed and stained tissue (Fig. 5a), and each site was assigned a laminar identity. We measured the peak amplitude of all of the EPSCs occurring within 150 ms after the stimulation and constructed colour-coded, linearly interpolated maps using the total amplitude of the EPSCs at each stimulation site. To quantify laminar input strength, we also calculated the normalized strength of the excitatory inputs from each layer by the summed values from all the layers examined. The data were analysed using custom software written in Matlab.

### Data and statistics

Data are presented as means±s.e. unless otherwise noted. Means were compared using the unpaired or paired *t*-test as indicated. Multiple comparisons were made using one-way ANOVA test, followed by a *post-hoc* Schaffe or Turkey's or Bonfferoni test. The variance of each data set were similar between groups; *P<0.05 and ***P<0.001. Although multiple dendrites were imaged in a single animal, the values for each animal were averaged and quoted sample sizes as used for statistical analysis represent number of different animals.

### Data availability

The authors declare that the data supporting the findings of this study are available within the article and its Supplementary Information files.

## Additional information

**How to cite this article:** Miyamoto, A. *et al*. Microglia contact induces synapse formation in developing somatosensory cortex. *Nat. Commun.* 7:12540 doi: 10.1038/ncomms12540 (2016).

## Supplementary Material

Supplementary Information Supplementary Figures 1-2

Supplementary Movie 1 Filopodia formation initiated by microglia contact onto a dendrite. The movie shows a microglial process in the somatosensory cortex of a P9 mouse approaching and then contacting a dendrite, after which a filopodia is formed. XYZ imaging was performed every 1 minute, with the total imaging time being 21 minutes. Red indicates a L2/3 pyramidal neuron dendrite, green indicates microglia. The images in Figure 1 are derived from this movie.

Supplementary Movie 2 Actin accumulation after microglia contact onto the dendrite. Time lapse *in vitro* imaging of actin accumulation after microglia contact in a cortical slice culture transfected with pCMV-lifeact-mCherry. XYt imaging was performed every 1.6 seconds. Total imaging time is 27 minutes. Red indicates F-actin in L2/3 pyramidal neuronal dendrite. Green indicates microglia. Scale bar is shown in Figure 2E, which derives from this movie.
