# Supplementary material for: Microglia contact induces synapse formation in developing somatosensory cortex
Source: Nat Commun. 2016 Aug 25;7:12540. doi: 10.1038/ncomms12540 (PMC5007295; doi:10.1038/ncomms12540)
Supplement: Supplementary Information — Supplementary Figures 1-2 [file ncomms12540-s1.pdf]

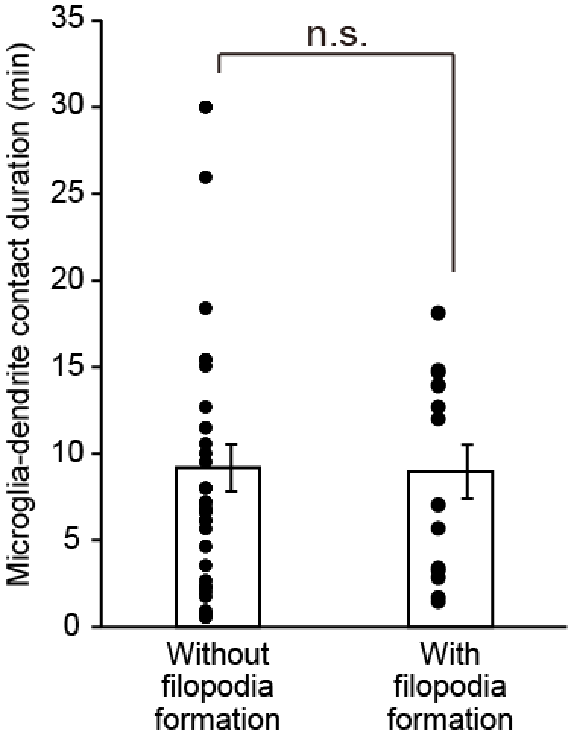

Supplementary Figure 1 Duration of microglial-dendrite contacts in P8-10 mice.

Bar graphs plotting the distribution of microglia-dendrite contact times for contacts that were followed by filopodia formation and for contacts that were not followed by filopodia formation. There was no difference in contact durations between these two groups.

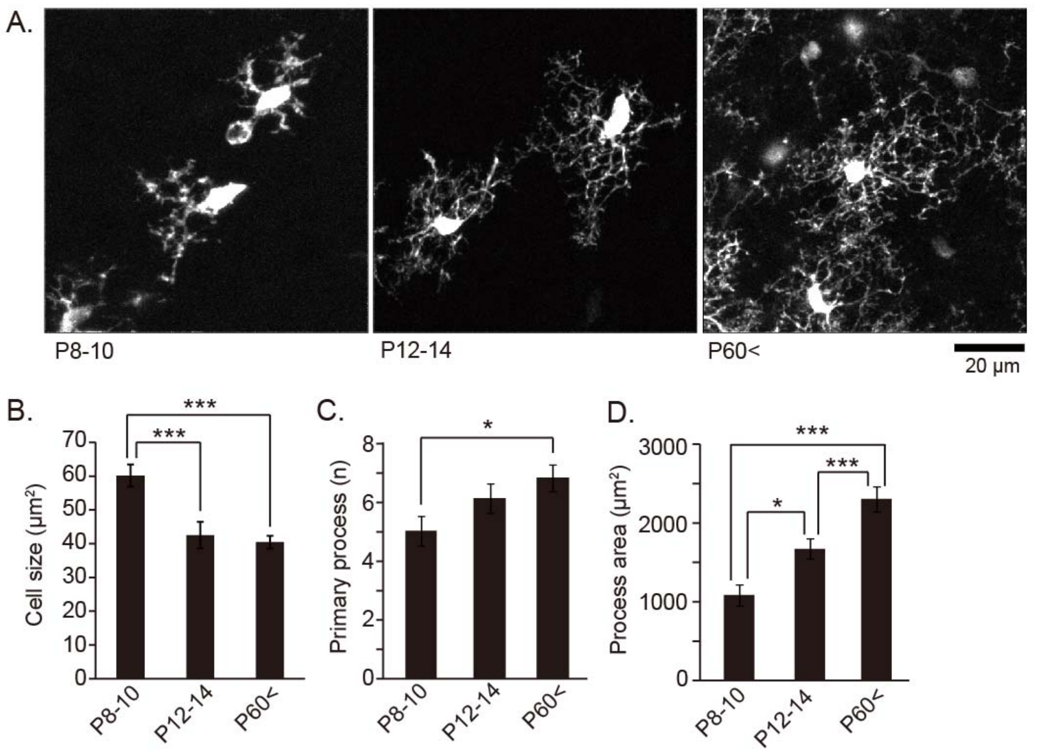

Supplementary Figure 2 Microglia have an activated morphology at P8-P10.

A. Representative *in vivo* images of microglia in L2/3 somatosensory cortex in Iba1-EGFP mice at different ages, illustrating the developmental change in microglia morphology from an amoeboid to ramified morphology. Scale bar 20 μm.

B. Quantification of microglial morphological parameters in three different developmental stages. The mean area of microglia soma decreased across development, from P8-P10 (60.2±3.3 μm^2), to P12-P14 (42.6±3.9 μm^2) and P>60 (40.5±1.9 μm^2)

C. The number of primary processes increased over development from P8-10 (5.0±0.5) through P12-P14 (6.1±0.5) to P>60 (6.8±0.5)

D. The area occupied by the microglial processes, as a measure of the extent of ramification, also

increased over development, from P8-P10 (1111.4±135.5 μm^2) to P12-P14 (1715.1±128.3 μm^2) and P>60 (2360.1±159.4 μm^2). The number of microglia analyzed were P8-P10 (11 microglia, from 3 mice), P12-P14 (12 microglia, from 3 mice) and P>60 (14 microglia, from 7 mice). ***P < 0.001, *P < 0.05, one-way ANOVA, post hoc: Scheffe. All data represent , mean±SEM
